# EspEn Graph for the Spatial Analysis of Entropy in Images

**DOI:** 10.3390/e25010159

**Published:** 2023-01-12

**Authors:** Ricardo Alonso Espinosa Medina

**Affiliations:** 1 Doctoral Program in Biomedical Engineering, University of Zaragoza, 50018 Zaragoza, Spain; respinosam@ecci.edu.co; 2 Department of Biomedical Engineering, Universidad ECCI, Bogotá 111311, Colombia

**Keywords:** entropy, irregularity, image, EspEn Graph, image processing

## Abstract

The quantification of entropy in images is a topic of interest that has had different applications in the field of agronomy, product generation and medicine. Some algorithms have been proposed for the quantification of the irregularity present in an image; however, the challenges to overcome in the computational cost involved in large images and the reliable measurements in small images are still topics of discussion. In this research we propose an algorithm, EspEn Graph, which allows the quantification and graphic representation of the irregularity present in an image, revealing the location of the places where there are more or less irregular textures in the image. EspEn is used to calculate entropy because it presents reliable and stable measurements for small size images. This allows an image to be subdivided into small sections to calculate the entropy in each section and subsequently perform the conversion of values to graphically show the regularity present in an image. In conclusion, the EspEn Graph returns information on the spatial regularity that an image with different textures has and the average of these entropy values allows a reliable measure of the general entropy of the image.

## 1. Introduction

Image processing allows us to obtain quantitative data to perform detection, recognition, segmentation and classification tasks. These tasks are useful in the generation of high-quality products and to reduce time and costs in the production of goods and services in different fields of industry, commerce, agriculture and medicine [1].

Currently there is great interest in objectively evaluating entropy in 2D data. Da Silva et al., in 2014, propose a two-dimensional sample entropy analysis (SampEn2D), an algorithm to classify groups of old and young rats through measures of irregularity obtained from histological images. The old rat images were more regular than the young rat images [2]. Subsequently, Da Silva et al., in 2016, used SampEn2D in three sets of images: simulated images, a database of images with different textures and biological images of rat sural nerves. The obtained measures of irregularity were reliable for images of relatively large size, but the measures were less reliable with images of small size. The properties and parameters of SampEn2D are two-dimensional matrix (u), patterns of length mxm, which are square windows (m), and tolerance threshold (r) [3]. Other algorithms to evaluate the entropy in images are Shannon’s entropy, described as the amount of individual information weighted by the probability of occurrence of the elements of the image [4], Distribution Entropy (DistrEn2D) and Dispersion Entropy (DispEn2D) [5], with interesting results, but focused on small images. Furthermore, mixing random values with an image does not significantly change the value of DistrIn2D [6]. Espinosa et al., in 2021, propose EspEn, an algorithm to quantify the regularity in both large and small images with great stability. However, the computational cost for large images is high and requires extensive computation time [7].

The challenge that arises in the calculation of entropy (image regularity) is related to the size of the images and the reliability of the measures of regularity obtained. In addition, entropy measures provide a single measure to evaluate the whole image, but there is no reliable algorithm that spatially shows the places in the image where there is more or less regularity.

In this study we propose an algorithm called EspEn Graph to measure the irregularity present in an image. The proposed algorithm sectorizes the total image to calculate the entropy, through EspEn, to show graphically the places where there is more or less entropy with a color code. This algorithm was applied to images from a database containing different textures.

## 2. Entropy Images

In this section, we expose the attempts of some investigations to show entropy graphically. Currently, much of the information we obtain is visual through cameras attached to drones, smartphones, security systems, laptops and other specialized devices such as robots. Image processing systems to extract information include artificial intelligence (AI) algorithms. These AI algorithms frequently extract features from the image to achieve better image classification or detection. [8].

Gen-Min Lin et al., in 2018, used entropy images to represent the complexity of fundus photographs, in order to improve performance in classifying diabetic retinopathy (DR) lesions using deep learning (neural network convolutional (CNN)). Entropy images increased the heterogeneity of fundus photographs and strengthened the contrast between RD lesions and unaffected areas. The spatial entropy used in this study is a function of the probability distribution of the local gray values [9]. The local entropy image is described as:

(1) $$E_{local}=-\underset{i}{\sum }P\left(i\right)\times log_{2}P\left(i\right)$$

In the probability density function, *P*(*i*) denotes the relative frequency associated with the *i*-th gray level within a *n* × *n* block [9]. This method is the application of Shannon Entropy in *n* × *n* sections (or windows) of an image.

Khattak et al., in 2015, analyzed the appearance and conditions of different skin lesions, which constitute a challenge for the development of better methods of segmentation of the affected area with respect to healthy tissue. The entropy image was made by calculating the maximum entropy based on Shannon et al.; for details of the calculation, see [10].

Ricardo Espinosa et al., in 2021, proposed the EspEn to evaluate the global entropy in an image. EspEn presented important advantages, compared to other similar algorithms (Shannon, SampEn2D, DistrEn2D, DispEn2D, among others), in relation to the stability of the measurement when the size of the image is smaller, and the sensitivity of the algorithm to determine the degree of contamination of an image [7]. Next, the EspEn algorithm and the EspEn Graph proposal are described.

### 2.1. EspEn Algorithm for Two Dimensions [7]

EspEn is an estimator of the irregularity of an image that considers the probability of occurrence of a set of samples, of dimension$\;m^{2}$, that are similar within a similarity threshold r, with an acceptable percentage in the number of samples similar [7]. The EspEn algorithm considers an image *u*(*i*,*j*) with width W and height H. Let *x_m_*(*i,j*) be the set of pixels that form a square window, with column range *j* to *j* + *m* − 1 and row range *i* to *i + m −* 1. The window construction would be *x_m_*(*i,j*) = [*u*(*i,j*)*, u*(*i, j +* 1), …, *u*(*i,j + m −* 1), *u*(*i +* 1,*j*), *u*(*i +* 1, *j +* 1), …, *u*(*i +* 1, *j + m −* 1), …, *u*(*i + m −* 1, *j + m −* 1)]. Then, EspEn is defined by the following:

(2) $$EspEn\left(u,m,r\right)=-\ln(D^{m})$$

where

(3) $$D^{m}=\frac{1}{\left(H-m+1\right)\left(W-m+1\right)}\overset{i=H-m+1;j=W-m+1}{\underset{i=1;j=1}{\sum }}C_{i,j}^{m}$$

(4) $$C_{i,j}^{m}=\frac{\left[\#\;of\;\varphi \left(r\right)\geq \rho \right]}{\left(H-m+1\right)\left(W-m+1\right)-1}$$

where ρ is fixed and represents the percentage of similarity acceptable for the study, expressed in decimals.

(5) $$\varphi \left(r\right)=\frac{[\#\;of\;x_{m}\left(a,b\right)|d[x_{m}\left(i,j\right),x_{m}\left(a,b\right)]\leq r]}{m^{2}}$$

where 1 ≤ *a* ≤ *H − m +* 1, 1 ≤ *b* ≤ *W* − *m* + 1 y (*a,b*) ≠ (*i,j*) to exclude self-matches. The distance function, *d*, for EspEn is defined by the following:

(6) $$d\left[x_{m}\left(i,j\right),x_{m}\left(a,b\right)\right]=\left|u\left(i+k,j+l\right)-u\left(a+k,b+l\right)\right|$$

where *k*, y, and *l* vary from 0 to *m* − 1.

### 2.2. Algorithm to Measure Entropy Graphically (EspEn Graph)

The EspEn presents great stability in entropy measurements when the images are of small size [7]. This feature is used to evaluate the entropy in small sections of an image.

**Figure 1:**
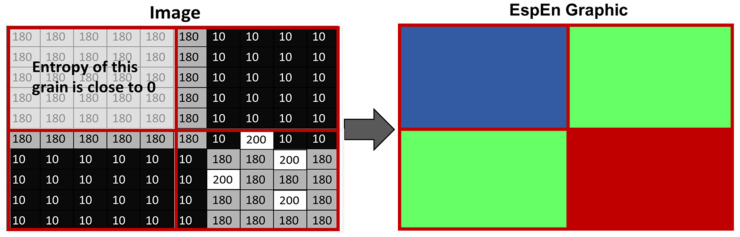
Representation of the calculation of the EspEn in a grain of the algorithm of the EspEn Graph. The red color represents greater irregularity, the blue color represents greater regularity, green colors (more yellow) represent intermediate values of irregularity.

The image is evenly divided into boxes called “grains”. Each grain is made up of a number of N × N pixels. The entropy in each grain is measured and the values obtained from the EspEn calculation (from 0 to approx. 10) are readjusted to integer values from 0 to 255, where values close to 0 represent a regular image, and values close to 255 represent an irregular image. Figure 1 shows a representation of an image with grains of 5 × 5 pixels whose entropy has subsequently been calculated. The entropy values of each grain are represented in a gray scale, where dark colors represent a regular image and light colors represent an irregular image, modifying the color map; the resulting image would be: blue colors represent a regular image, red represent an irregular image and intermediate values of entropy is shown by green, yellow and orange colors.

## 3. Materials and Methods

### 3.1. Image Set

Synthetic images with repetitive (predictable) and clearly identifiable patterns (shapes) were created, following the methodology described in [7]. These images were progressively contaminated with uniform white noise, similar to the process shown with detailed MIX2D in [3], defined as:

(7) $$MIX{\left(p\right)}_{ij}=\left(1-p\right)X_{ij}+p\;Y_{ij}$$

where *X_ij_* is the synthetic image, *Y_ij_* the noise image with uniform distribution and *p* represents the degree of contamination: *p* = 0 (no contamination) and *p* = 1 (only noise). Four images with different degrees of contamination were obtained: MIX(0), MIX(0.33), MIX(0.66), and MIX(1). From each MIX image, ¼ fraction was taken to create a new image, in different distributions. Figure 1Figure 3a–c show the new images obtained, called ImMIX.

**Figure 2:**
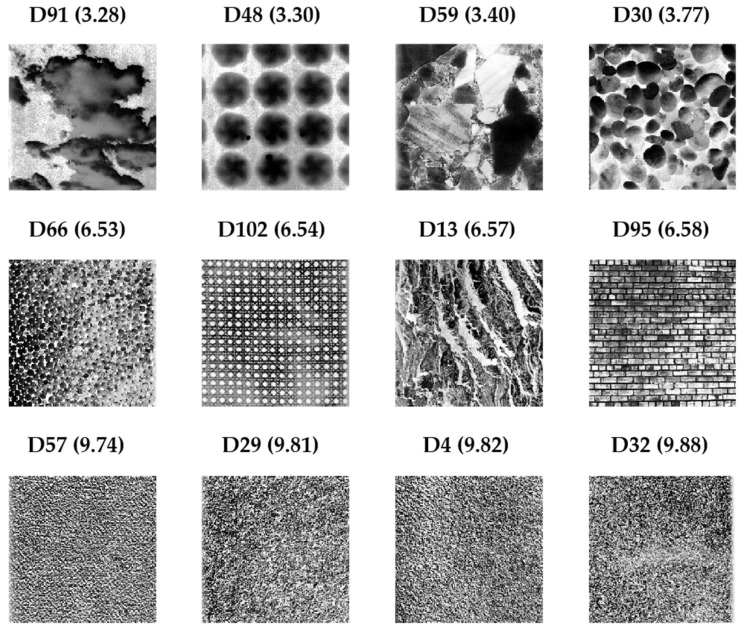
Images from the normalized Brodatz texture database (NBT) used in the second numerical experiment. Top row regular images (low entropy value), middle row medium irregular images (middle entropy value) and lower row irregular images (high entropy value).

A second set of images from the normalized Brodatz texture database (NBT) was used, which contains normalized images with different textures; the details of the characteristics of this database can be seen in [7]. NBT images with low (approx. 3.4), medium (approx. 6.5) and high (approx. 9.8) entropy values quantified with EspEn [7] were used. Figure 2 shows the NBT images used with their respective denomination and entropy value calculated with EspEn, reported in [7]. Fractions of the NBT images were taken to create images with varied textures: a group of images with half of the image with regular texture (predominant fraction), a quarter of the image completely irregular and the remaining quarter with a half irregular texture. In the same way, a second group of images with a predominance of medium irregular texture and a third group of images with an irregular predominance were prepared. We call these image sets ImNBT in the following sections.

**Figure 3:**
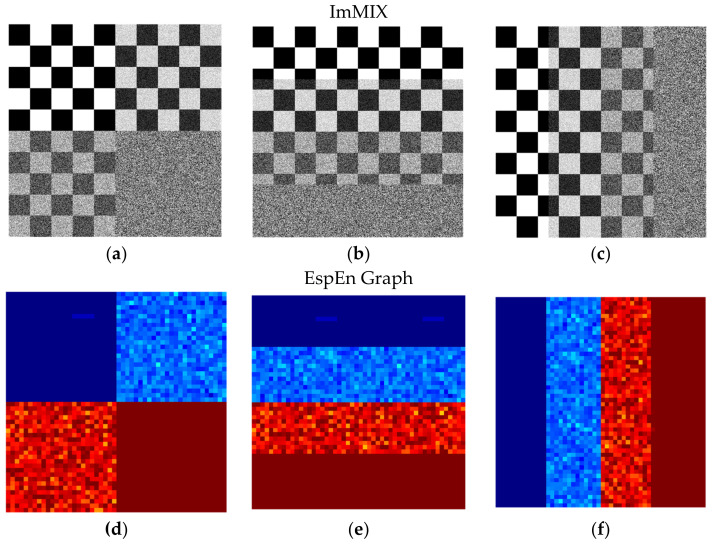
Synthetic images with homogeneous fractions of regular and irregular shapes (ImMIX (**a**–**c**)). Result of the EspEn Graph for each case (**d**–**f**), shows the most irregular areas in red, the most regular areas in blue, and the areas with intermediate degrees of contamination in orange and light blue. The distribution pattern of the original images with different sections (**a**–**c**) is maintained in the results (**d**–**f**).

### 3.2. Experiment and Parameters

Two numerical experiments were carried out to evaluate the capacity of the EspEn Graph; the first experiment evaluates the stability of the measurements when the sizes of the image are varied and the second experiment evaluates the capacity of regular and irregular classification of an image with different textures inside.

The first experiment consisted of applying the EspEn Graph to obtain a new image with information on the spatial entropy of the image. ImMIX images were upsampled (s = 2, 5, and 10) to change image size. The original image is 500 × 500 pixels and the sampled images have dimensions of 250 × 250, 100 × 100 and 50 × 50 pixels for s = 2, 5 and 10, respectively.

In addition, the overall EspEn of each sampled ImMIX image and the mean of the EspEn entropy values of all the grains in each sampled image were calculated to compare the EspEn values with the average value of EspEn Graph. The parameters used in the EspEn algorithm were the image (u), the length of the square window (m = 3), the percentage of similarity between windows (ρ = 0.7) and the similarity threshold (r = 35). In addition to these values, for the EspEn Graph, the dimensions of the “grain” were set at 10 × 10.

The second experiment consisted in calculating the average of the EspEn Graph in ImNBT images to demonstrate that it allows the classification of regular and irregular images, when they have a regular or irregular predominance, respectively.

## 4. Results

The calculation of entropy in images is a topic of interest today. EspEn has proven to be a robust algorithm for measuring entropy in large and small images while maintaining a certain stability in the measurement. In this study, an algorithm has been proposed to measure the EspEn graphically, because the images can contain different shapes and textures and a single general measurement of the entropy is not enough to obtain detailed information on the regularity within the image.

Figure 3 shows the entropy image returned by the EspEn Graph algorithm for each of the images (Figure 3a–c) that have different sections with different degrees of contamination. The resulting images (Figure 3d–f) show the areas of greatest irregularity in red, the more regular areas in blue and the areas with intermediate degrees of contamination in orange and light blue. The EspEn Graph correctly delimits areas with different degrees of irregularity; this capability is important for segmentation applications based on entropy.

**Figure 4:**
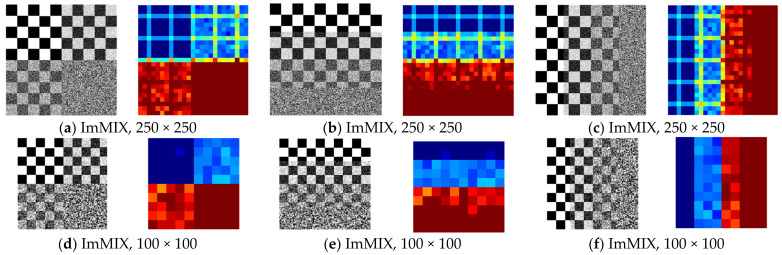

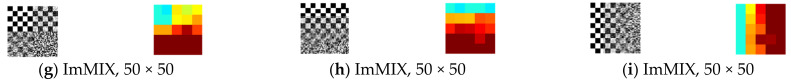
Result of the EspEn Graph for ImMIX images with different spatial distribution of noise contamination, in equal amounts and different sizes. Color images (**a**–**c**) show greater detail in the identification of regularity through colors (red = irregular, dark blue = regular, light blue = 33% contamination and orange = 66% contamination). The color images (**d**–**f**) show a clear differentiation of the regularity, with less detail, and finally the color images (**g**–**i**) show a differentiation of the regularity of the image that is not very detailed but identifiable.

Table 1 shows the values of the general entropy (EspEn) calculated for the images in gray ImMIX (Figure 4a–c). These images have spatially distributed the same amount of a regular image fraction, a regular image contaminated with noise at 33% and 66%, and finally a noise-only fraction. The EspEn remained similar for all images in gray ImMIX (Figure 4a–i), even when the images were resized. The mean of the entropy values, when the EspEn Graph was applied to the ImMIX gray images (Figure 4a–i), turned out to be similar for images with the same size, but different when the size of the images was changed. The most relevant information of the EspEn Graph is visual, because it allows spatial identification of the places in the image where there is more or less regularity.

Figure 4 shows the result of the EspEn Graph for each ImMIX with different sizes. The identification of the regular and irregular regions was maintained in all cases, both for large and small images. The EspEn Graph shows in a stable way the colors that characterize the irregularity in the images when they have different sizes.

**Figure 5:**
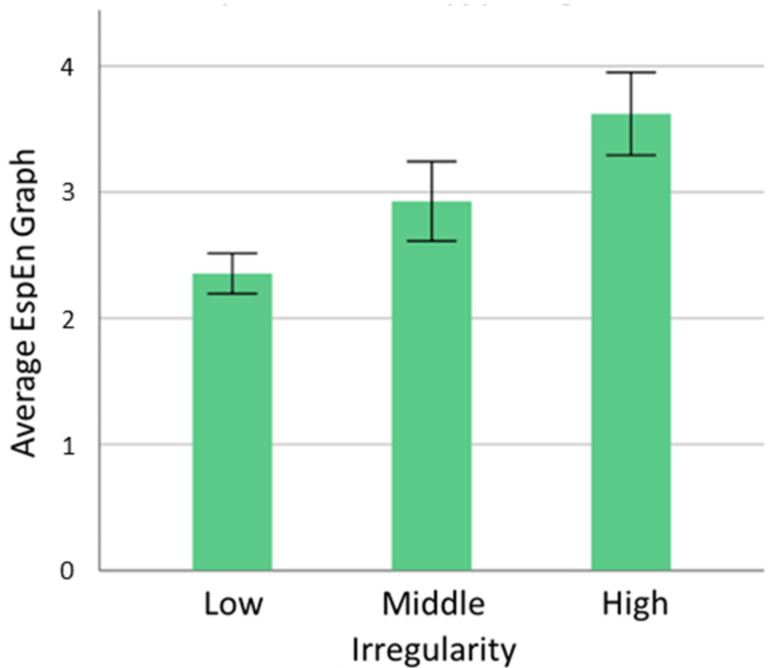
Mean ± std of the EspEn Graph for the different groups of ImNBT images.

Figure 5 shows the graph of the averages of the EspEn Graph where the separation between ImNBT images with different predominance of irregularity (low, intermediate and high) is evidenced. In addition, the average of the values of the EspEn Graph can be used to estimate the global entropy of an image in less time and computational cost compared to EspEn.

**Figure 6:**
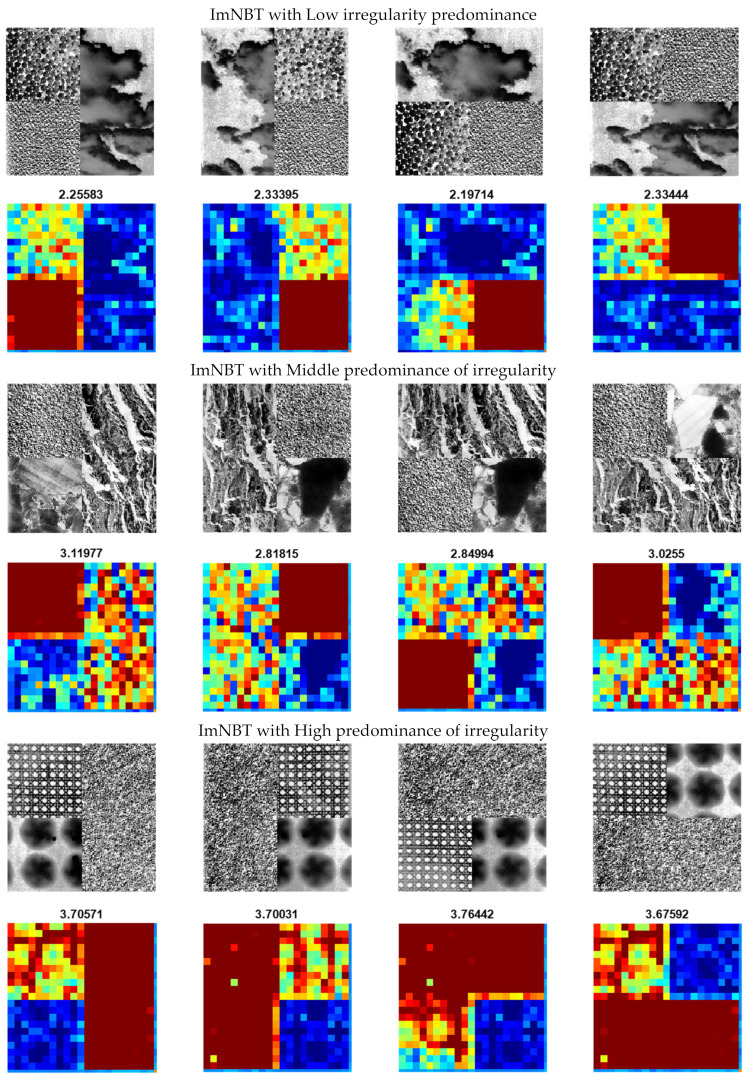
ImNBT images with different textures and different irregularity predominance (low, middle and high) and their respective EspEn Graph images with the mean values of the EspEn Graph entropy.

Figure 6 shows the result of the EspEn Graph for the ImNBT images with different predominance of regular and irregular textures. It shows, as examples, only one image with a predominance of regularity, middle regularity and irregularity with low, intermediate and high entropy values respectively, which occupy half of the total image in four different spatial distributions.

## 5. Application of the EspEn Graph

The estimation of entropy in images is a very useful tool in medicine, technology and industry. The EspEn Graph method, proposed in this study, shows the regularity in different sections of the image. Next, some investigations that use entropy in images are presented, the result of these investigations providing relevant information on the characteristics of the material, structure or surface under study. In addition, the potential use of EspEn Graph in these works for future research is exposed.

Miao et al., in 2019, studied the surface degradation of different functional road pavements for two years. The evaluation of the deterioration of the pavement structure was carried out through the entropy applied to images, each one with different asphalt textures. The results showed the distinction between smooth pavements, described through a low entropy value, compared to rougher pavements, with high entropy values. This finding demonstrated advantages for evaluating the anti-slip characteristics of pavement macrostructures [11].

The EspEn Graph, for the evaluation of pavement regularity could show the degradation of different types of paving materials when different pavement structures are shown in the same image; an example is the filling of a hole in a section of the road. The EspEn Graph facilitates the identification of the entropy of different materials in the same graph, unlike the method used in the work of [11].

Fastowicz et al., in 2019, evaluated the regularity of the surface of 3D printed parts, through the estimation of the regularity in images of 3D scanners applied to the printed parts, in order to determine the quality of the surfaces during printing [12].

The application of the EspEn Graph would show the precise place where the printing defect is located and the average of the values of the EspEn Graph would indicate the general quality of the surface of the printed parts. Locating the specific place of the 3D printing defect can help determine the conditions that are met in the printing environment and consequently allows to correct in advance the events that influence achievement of a high-quality print, avoiding waste of material and loss of time in printing.

Wu et al., in 2013, calculated the entropy of different sections of an image that do not overlap, to evaluate encrypted images, overcoming the weaknesses shown (inaccuracy, inconsistency and low efficiency) when evaluating the quality of encryption using global entropy [13].

In an encrypted image, it is important that it does not have recognizable patterns in order to guarantee the security of the information. For this reason, evaluating the encryption efficiency in an image allows greater security and the possibility of improving current encryption systems. The application of EspEn Graph in this type of study would allow us to indicate the precise place where patterns are found in the encrypted image and, in general, would show the quality of the encryption.

Breslavets et al., in 2019, evaluated the severity of a skin lesion by quantifying the entropy in images of healthy and damaged skin. In medical practice, the evaluation of skin lesions is subjective and can change from evaluator to evaluator, so it is important to propose an objective tool for quantitative skin analysis [14].

The use of EspEn Graph in images of the skin can help determine the degree of skin lesion and the place of greatest involvement of the lesion; since skin lesions are not normally uniform, it is important to delimit and quantify places of greater or less gravity.

## 6. Conclusions

The algorithms to estimate the irregularity in images have been very useful for the analysis, classification or segmentation of textures. However, a single entropy measure for an image is not enough to detail the regions of greater or lesser regularity present in the same image. The EspEn Graph returns an image showing the regions of greater or lesser regularity graphically in gray scale or with a map of different colors. This study shows that EspEn Graph is capable of recognizing the irregularity present in any region of the image of any size. Although the entropy measures could vary by changing the size of the image, if the comparison is made between images of the same size with different degrees of irregularity, the entropy measure is very consistent. The EspEn Graph is sensitive to the predominance of the irregularity present in an image, showing not only graphically the place of greater or lesser regularity, but the mean of the EspEn Graph values could be a measure of the total entropy of the image with a significant decrease in computational cost, because it calculates the entropy in small sections of the image and does not make extensive comparisons with each pixel of the image, which can be computationally extensive with large images. EspEn Graph is presented as a robust tool for calculating entropy with potential applications in industry, commerce, agronomy and medicine.

## Figures and Tables

**Table 1:** EspEn values and the mean of the entropy values of the EspEn Graph.

Images	Sampling (s)	Dimensions	EspEn	Mean EspEn Graph
ImMIX(a)	2	250 × 250	4.0376	3.0734
ImMIX(b)	2	250 × 250	4.0823	3.1322
ImMIX(c)	2	250 × 250	4.0906	3.1675
ImMIX(a)	5	100 × 100	4.1870	2.8370
ImMIX(b)	5	100 × 100	4.2190	2.9302
ImMIX(c)	5	100 × 100	4.2190	2.9590
ImMIX(a)	10	50 × 50	4.9705	4.0916
ImMIX(b)	10	50 × 50	4.9377	4.2453
ImMIX(c)	10	50 × 50	4.9377	4.1169

## Data Availability

Not applicable.
